# The genome sequence of the Basking Shark, *Cetorhinus maximus* (Gunnerus, 1765) (Lamniformes: Cetorhinidae)

**DOI:** 10.12688/wellcomeopenres.26114.1

**Published:** 2026-03-18

**Authors:** Nicholas J. Davison, Wellcome Sanger Institute Tree of Life Management, Samples and Laboratory team, Wellcome Sanger Institute Scientific Operations: Sequencing Operations, Wellcome Sanger Institute Tree of Life Core Informatics team, Tree of Life Core Informatics collective, Darwin Tree of Life Consortium

**Affiliations:** 1 Scottish Marine Animal Stranding Scheme (SMASS), School of Biodiversity, One Health and Veterinary Medicine, University of Glasgow College of Medical Veterinary and Life Sciences, Glasgow, Scotland, UK

**Keywords:** Cetorhinus maximus; Basking Shark; genome sequence; chromosomal; Lamniformes

## Abstract

We present a genome assembly from an individual female *Cetorhinus maximus* (Basking Shark; Chordata; Chondrichthyes; Lamniformes; Cetorhinidae). The assembly contains two haplotypes with total lengths of 3 993.85 megabases and 3 817.33 megabases. Most of haplotype 1 (88.16%) is scaffolded into 39 chromosomal pseudomolecules, including the X sex chromosome. Haplotype 2 was assembled to scaffold level. The mitochondrial genome has also been assembled, with a length of 16.67 kilobases. This assembly was generated as part of the Darwin Tree of Life project, which produces reference genomes for eukaryotic species found in Britain and Ireland.

## Species taxonomy

Eukaryota; Opisthokonta; Metazoa; Eumetazoa; Bilateria; Deuterostomia; Chordata; Craniata; Vertebrata; Gnathostomata; Chondrichthyes; Elasmobranchii; Selachii; Galeomorphii; Galeoidea; Lamniformes; Cetorhinidae; *Cetorhinus*; *Cetorhinus maximus* (Gunnerus, 1765) (NCBI:txid57982)

## Background

The basking Shark ( *Cetorhinus maximus*) is the second largest fish only extant member of the family *Cetorhinidae* ( Sims, 2008). It is a coastal-pelagic shark found worldwide in boreal to warm-temperate waters. It is commonly found around the continental shelf and occasionally enters brackish waters ( Wilson & Wilding, 2017). It is one of only three sharks that filter feed. These sharks follow plankton concentrations in the water column, and is often visible at the surface in the summer months around the British Isles. The basking shark regularly reaches 7–8.5 m in length with some individuals reaching 12 m and a weight of 4000 kg ( Sims, 2008). Their large size, very large gill slits that virtually incircle the head, dermal denticle gillrakers, pointed snout, large, subterminal mouth with minute hooked teeth, caudal peduncle with strong lateral keels, and lunate caudal fin distinguish this shark from all others. The basking shark feeds exclusively on small planktonic organisms trapped on its unique gillrakers. They are ovoviviparous. It is thought that the female continues to produce infertile eggs during pregnancy which the embryos can feed on ( Compagno, 1984). However, ( Ali *et al.*, 2012) suggested that oophagy would not be possible due to the large size of the egg capsules and the planktonic feeding method of the basking shark. Estimated gestation period have resulted in a broad time scale, from one to 3.5 years, after which, about six pups are born ( Sims, 2008; Sund, 1943).

It is listed as endangered globally by the IUCN, with a decreasing population. Although no longer targeted, it is still caught as bycatch in trawl, trammel nets, and set-net fisheries, and becomes entangled in pot lines. The large fins are extremely valuable in the fin trade. Across regions, there have been some severe historic declines, however there are indications of some stability and possible slow recovery since cessation of target fishing and high levels of protection. The global population may now be beginning to stabilise, with signs of that from the Northeast Atlantic, although elsewhere there is little information upon which is assess stability. However, abundances are still estimated to be well below historic levels and there is ongoing demand for the high-value fins ( IUCNredlist.org, consulted 20 November 2025).

**Figure 1.**
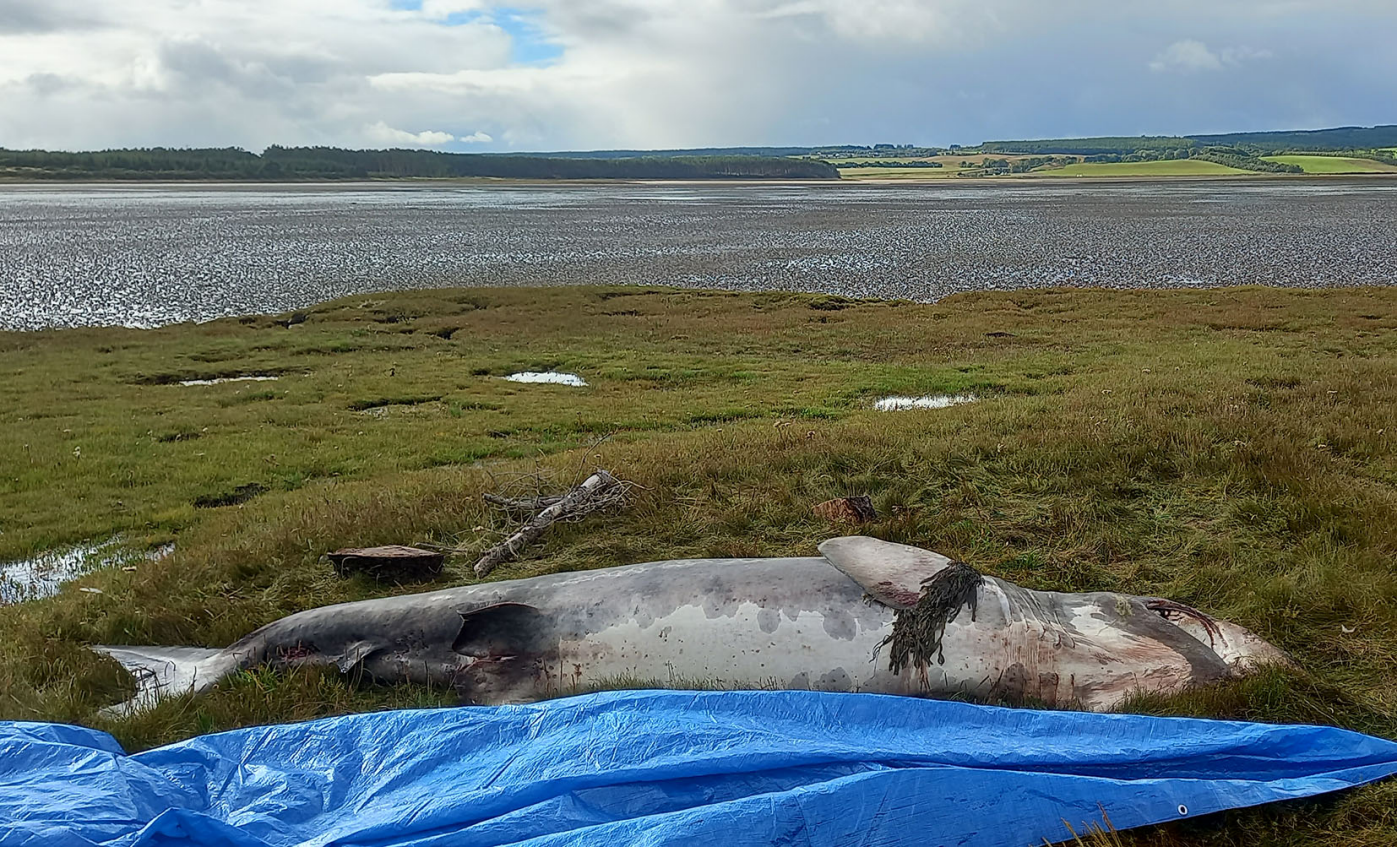
Photograph of the *Cetorhinus maximus* (sCetMax3) carcass from which samples were taken for genome sequencing.

The assembly was produced using the Tree of Life pipeline from a specimen (M489/22) ( Figure 1), collected at Loch Fleet, Highland, Scotland, United Kingdom. This is the first publicly available genome for the genus *Cetorhinus* and for the family Cetorhinidae as of January 2026 (data obtained via NCBI datasets, O’Leary *et al.*, 2024). It was generated as part of the Darwin Tree of Life Project, which aims to generate high-quality reference genomes for all named eukaryotic species in Britain and Ireland to support research, conservation, and the sustainable use of biodiversity.

## Methods

### Sample acquisition

The specimen used for genome sequencing was a juvenile female *Cetorhinus maximus* (specimen ID SAN00002660, ToLID sCetMax3; Figure 1), collected from Loch Fleet, Highland, Scotland, United Kingdom (latitude 57.9502, longitude –4.0192) on 2022-09-15. The specimen was collected and identified by Nick Davison (Scottish Marine Animal Stranding Scheme University of Glasgow). The same specimen was used for RNA sequencing.

### Nucleic acid extraction

Protocols for high molecular weight (HMW) DNA extraction developed at the Wellcome Sanger Institute (WSI) Tree of Life Core Laboratory are available on protocols.io ( Howard *et al.*, 2025). The sCetMax3 sample was weighed and triaged to determine the appropriate extraction protocol. Tissue from the heart was homogenised by cryogenic disruption using the Covaris cryoPREP ^®^ Automated Dry Pulverizer. HMW DNA was extracted in the WSI Scientific Operations core using the Manual MagAttract v2 protocol. DNA was sheared into an average fragment size of 12–20 kb following the Megaruptor ^®^3 for LI PacBio protocol. Sheared DNA was purified by automated SPRI (solid-phase reversible immobilisation). The concentration of the sheared and purified DNA was assessed using a Nanodrop spectrophotometer and Qubit Fluorometer using the Qubit dsDNA High Sensitivity Assay kit. Fragment size distribution was evaluated by running the sample on the FemtoPulse system.

RNA was also extracted from heart tissue of sCetMax3 in the Tree of Life Laboratory at the WSI using the RNA Extraction: Automated MagMax™ *mir*Vana protocol. The RNA concentration was assessed using a Nanodrop spectrophotometer and a Qubit Fluorometer using the Qubit RNA Broad-Range Assay kit. Analysis of the integrity of the RNA was done using the Agilent RNA 6000 Pico Kit and Eukaryotic Total RNA assay.

### PacBio HiFi library preparation and sequencing

Library preparation and sequencing were performed at the WSI Scientific Operations core. Libraries were prepared using the SMRTbell Prep Kit 3.0 (Pacific Biosciences, California, USA), following the manufacturer’s instructions. The kit includes reagents for end repair/A-tailing, adapter ligation, post-ligation SMRTbell bead clean-up, and nuclease treatment. Size selection and clean-up were performed using diluted AMPure PB beads (Pacific Biosciences). DNA concentration was quantified using a Qubit Fluorometer v4.0 (ThermoFisher Scientific) and the Qubit 1X dsDNA HS assay kit. Final library fragment size was assessed with the Agilent Femto Pulse Automated Pulsed Field CE Instrument (Agilent Technologies) using the gDNA 55 kb BAC analysis kit.

The sample was sequenced on a Revio instrument (Pacific Biosciences). The prepared library was normalised to 2 nM, and 15 μL was used for making complexes. Primers were annealed and polymerases bound to generate circularised complexes, following the manufacturer’s instructions. Complexes were purified using 1.2X SMRTbell beads, then diluted to the Revio loading concentration (200–300 pM) and spiked with a Revio sequencing internal control. The sample was sequenced on a Revio 25M SMRT cell. The SMRT Link software (Pacific Biosciences), a web-based workflow manager, was used to configure and monitor the run and to carry out primary and secondary data analysis.

### Hi-C

***Sample preparation and crosslinking***

The Hi-C sample was prepared from 20–50 mg of frozen heart tissue from the sCetMax3 sample using the Arima-HiC v2 kit (Arima Genomics). Following the manufacturer’s instructions, tissue was fixed and DNA crosslinked using TC buffer to a final formaldehyde concentration of 2%. The tissue was homogenised using the Diagnocine Power Masher-II. Crosslinked DNA was digested with a restriction enzyme master mix, biotinylated, and ligated. Clean-up was performed with SPRISelect beads before library preparation. DNA concentration was measured with the Qubit Fluorometer (Thermo Fisher Scientific) and Qubit HS Assay Kit. The biotinylation percentage was estimated using the Arima-HiC v2 QC beads.

***Hi-C library preparation and sequencing***

Biotinylated DNA constructs were fragmented using a Covaris E220 sonicator and size selected to 400–600 bp using SPRISelect beads. DNA was enriched with Arima-HiC v2 kit Enrichment beads. End repair, A-tailing, and adapter ligation were carried out with the NEBNext Ultra II DNA Library Prep Kit (New England Biolabs), following a modified protocol where library preparation occurs while DNA remains bound to the Enrichment beads. Library amplification was performed using KAPA HiFi HotStart mix and a custom Unique Dual Index (UDI) barcode set (Integrated DNA Technologies). Depending on sample concentration and biotinylation percentage determined at the crosslinking stage, libraries were amplified with 10–16 PCR cycles. Post-PCR clean-up was performed with SPRISelect beads. Libraries were quantified using the AccuClear Ultra High Sensitivity dsDNA Standards Assay Kit (Biotium) and a FLUOstar Omega plate reader (BMG Labtech).

Prior to sequencing, libraries were normalised to 10 ng/μL. Normalised libraries were quantified again to create equimolar and/or weighted 2.8 nM pools. Pool concentrations were checked using the Agilent 4200 TapeStation (Agilent) with High Sensitivity D500 reagents before sequencing. Sequencing was performed using paired-end 150 bp reads on the Illumina NovaSeq X.

### RNA library preparation and sequencing

Libraries were prepared using the NEBNext ^®^ Ultra™ II Directional RNA Library Prep Kit for Illumina (New England Biolabs), following the manufacturer’s instructions. Poly(A) mRNA in the total RNA solution was isolated using oligo (dT) beads, converted to cDNA, and uniquely indexed; 14 PCR cycles were performed. Libraries were size-selected to produce fragments between 100–300 bp. Libraries were quantified, normalised, pooled to a final concentration of 2.8 nM, and diluted to 150 pM for loading. Sequencing was carried out on the Illumina NovaSeq X, generating paired-end reads.

### Genome assembly

Prior to assembly of the PacBio HiFi reads, a database of *k*-mer counts ( *k* = 31) was generated from the filtered reads using FastK. GenomeScope2 ( Ranallo-Benavidez *et al.*, 2020) was used to analyse the *k*-mer frequency distributions, providing estimates of genome size, heterozygosity, and repeat content.

The HiFi reads were assembled using Hifiasm in Hi-C phasing mode ( Cheng *et al.*, 2021, 2022), producing two haplotypes. Hi-C reads ( Rao *et al.*, 2014) were mapped to the primary contigs using bwa-mem2 ( Vasimuddin *et al.*, 2019). Contigs were further scaffolded with Hi-C data in YaHS ( Zhou *et al.*, 2023), using the --break option for handling potential misassemblies. The scaffolded assemblies were evaluated using Gfastats ( Formenti *et al.*, 2022), BUSCO ( Manni *et al.*, 2021) and MERQURY.FK ( Rhie *et al.*, 2020).

The mitochondrial genome was assembled using MitoHiFi ( Uliano-Silva *et al.*, 2023).

### Assembly curation

The assembly was decontaminated using the Assembly Screen for Cobionts and Contaminants ( ASCC) pipeline. TreeVal was used to generate the flat files and maps for use in curation. Manual curation was conducted primarily in PretextView and HiGlass ( Kerpedjiev *et al.*, 2018). Scaffolds were visually inspected and corrected as described by Howe *et al*. (2021). Manual corrections included 34 breaks and 245 joins. This reduced the scaffold count by 2.2%. The curation process is described at https://gitlab.com/wtsi-grit/rapid-curation . PretextSnapshot was used to generate a Hi-C contact map of the final assembly.

### Assembly quality assessment

The Merqury.FK tool ( Rhie *et al.*, 2020) was run in a Singularity container ( Kurtzer *et al.*, 2017) to evaluate *k*-mer completeness and assembly quality for both haplotypes using the *k*-mer databases ( *k* = 31) computed prior to genome assembly. The analysis outputs included assembly QV scores and completeness statistics.

The genome was analysed using the BlobToolKit pipeline, a Nextflow implementation of the earlier Snakemake version ( Challis *et al.*, 2020). The pipeline aligns PacBio reads using minimap2 ( Li, 2018) and SAMtools ( Danecek *et al.*, 2021) to generate coverage tracks. It runs BUSCO ( Manni *et al.*, 2021) using lineages identified from the NCBI Taxonomy ( Schoch *et al.*, 2020). For the three domain-level lineages, BUSCO genes are aligned to the UniProt Reference Proteomes database ( Bateman *et al.*, 2023) using DIAMOND blastp ( Buchfink *et al.*, 2021). The genome is divided into chunks based on the density of BUSCO genes from the closest taxonomic lineage, and each chunk is aligned to the UniProt Reference Proteomes database with DIAMOND blastx. Sequences without hits are chunked using seqtk and aligned to the NT database with blastn ( Altschul *et al.*, 1990). The BlobToolKit suite consolidates all outputs into a blobdir for visualisation. The BlobToolKit pipeline was developed using nf-core tooling ( Ewels *et al.*, 2020) and MultiQC ( Ewels *et al.*, 2016), with containerisation through Docker ( Merkel, 2014) and Singularity ( Kurtzer *et al.*, 2017).

## Genome sequence report

### Sequence data

**Figure 2.**
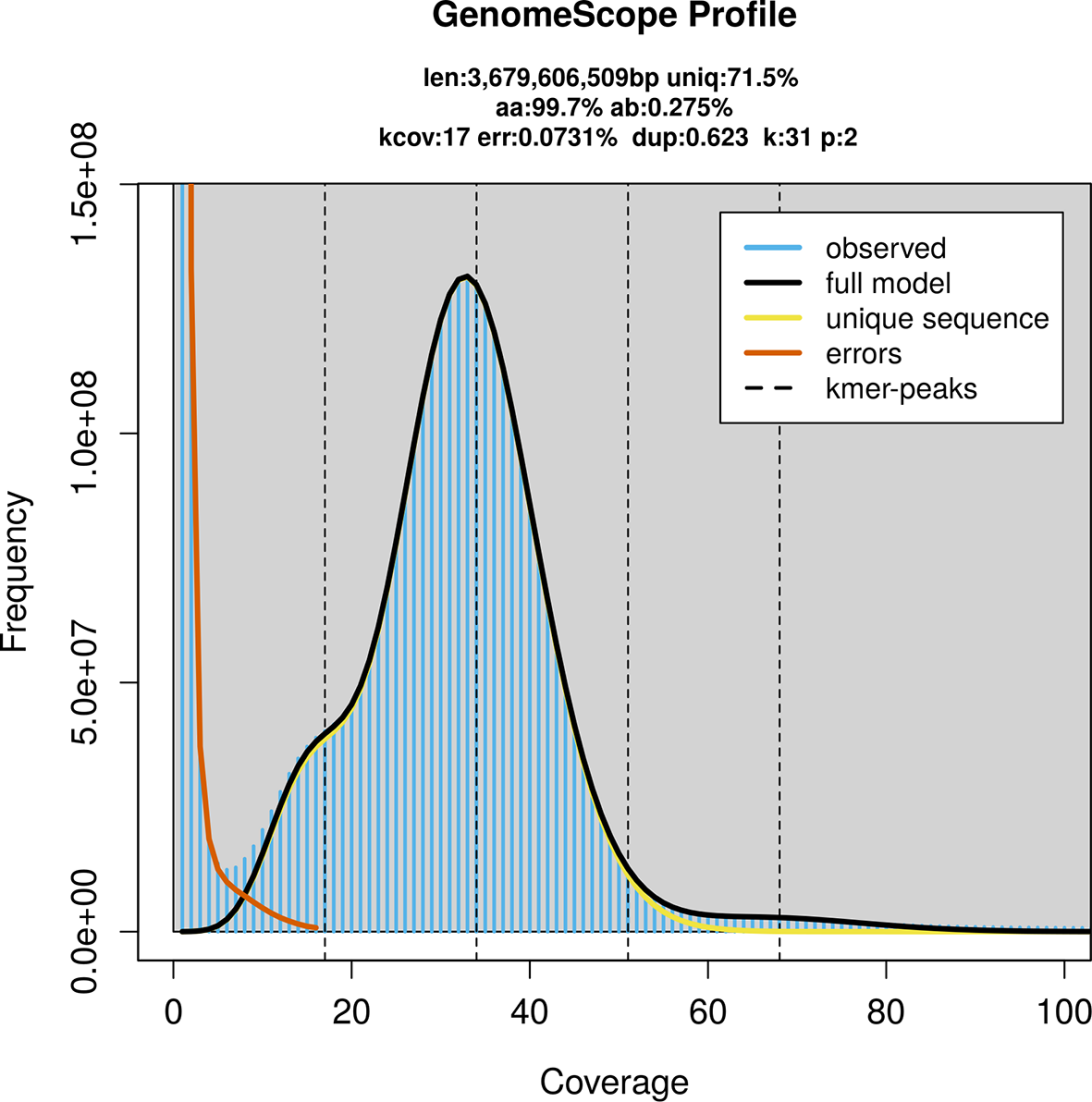
Frequency distribution of *k*-mers generated using GenomeScope2. The plot shows observed and modelled *k*-mer spectra, providing estimates of genome size, heterozygosity, and repeat content based on unassembled sequencing reads.

PacBio sequencing of the *Cetorhinus maximus* specimen generated 128.60 Gb (gigabases) from 18.62 million reads, which were used to assemble the genome. GenomeScope2.0 analysis estimated the haploid genome size at 3 679.61 Mb, with a heterozygosity of 0.27% and repeat content of 28.60% ( Figure 2). These estimates guided expectations for the assembly. Based on the estimated genome size, the sequencing data provided approximately 34× coverage. Hi-C sequencing produced 870.61 Gb from 5 765.61 million reads, which were used to scaffold the assembly. RNA sequencing data were also generated and are available in public sequence repositories. Table 1 summarises the specimen and sequencing details.

**Table 1.** Specimen and sequencing data for BioProject PRJEB75718.

Platform	PacBio HiFi	Hi-C	RNA-seq
**ToLID**	sCetMax3	sCetMax3	sCetMax3
**Specimen ID**	SAN00002660	SAN00002660	SAN00002660
**BioSample (source individual)**	SAMEA113902670	SAMEA113902670	SAMEA113902670
**BioSample (tissue)**	SAMEA113902671	SAMEA113902671	SAMEA113902671
**Tissue**	heart	heart	heart
**Instrument**	Revio	Illumina NovaSeq X	Illumina NovaSeq X
**Run accessions**	ERR13112082; ERR13112080; ERR13112081	ERR13132926; ERR13132927	ERR13132928
**Read count total**	18.62 million	5 765.61 million	38.86 million
**Base count total**	128.60 Gb	870.61 Gb	5.87 Gb

### Assembly statistics

The genome was assembled into two haplotypes using Hi-C phasing. Haplotype 1 was curated to chromosome level, while haplotype 2 was assembled to scaffold level. The final assembly has a total length of 3 993.85 Mb in 5 087 scaffolds, with 2 778 gaps, and a scaffold N50 of 126.03 Mb ( Table 2).

**Table 2.** Genome assembly statistics.

**Assembly name**	sCetMax3.hap1.1	sCetMax3.hap2.1
**Assembly accession**	GCA_964194155.1	GCA_964194165.1
**Assembly level**	chromosome	scaffold
**Span (Mb)**	3 993.85	3 817.33
**Number of chromosomes**	39	-
**Number of contigs**	7 865	6 569
**Contig N50**	1.96 Mb	2.09 Mb
**Number of scaffolds**	5 087	3 829
**Scaffold N50**	126.03 Mb	127.93 Mb
**Longest scaffold length (Mb)**	250.19	-
**Sex chromosomes**	X	-
**Organelles**	Mitochondrion: 16.67 kb	-

**Figure 3.**
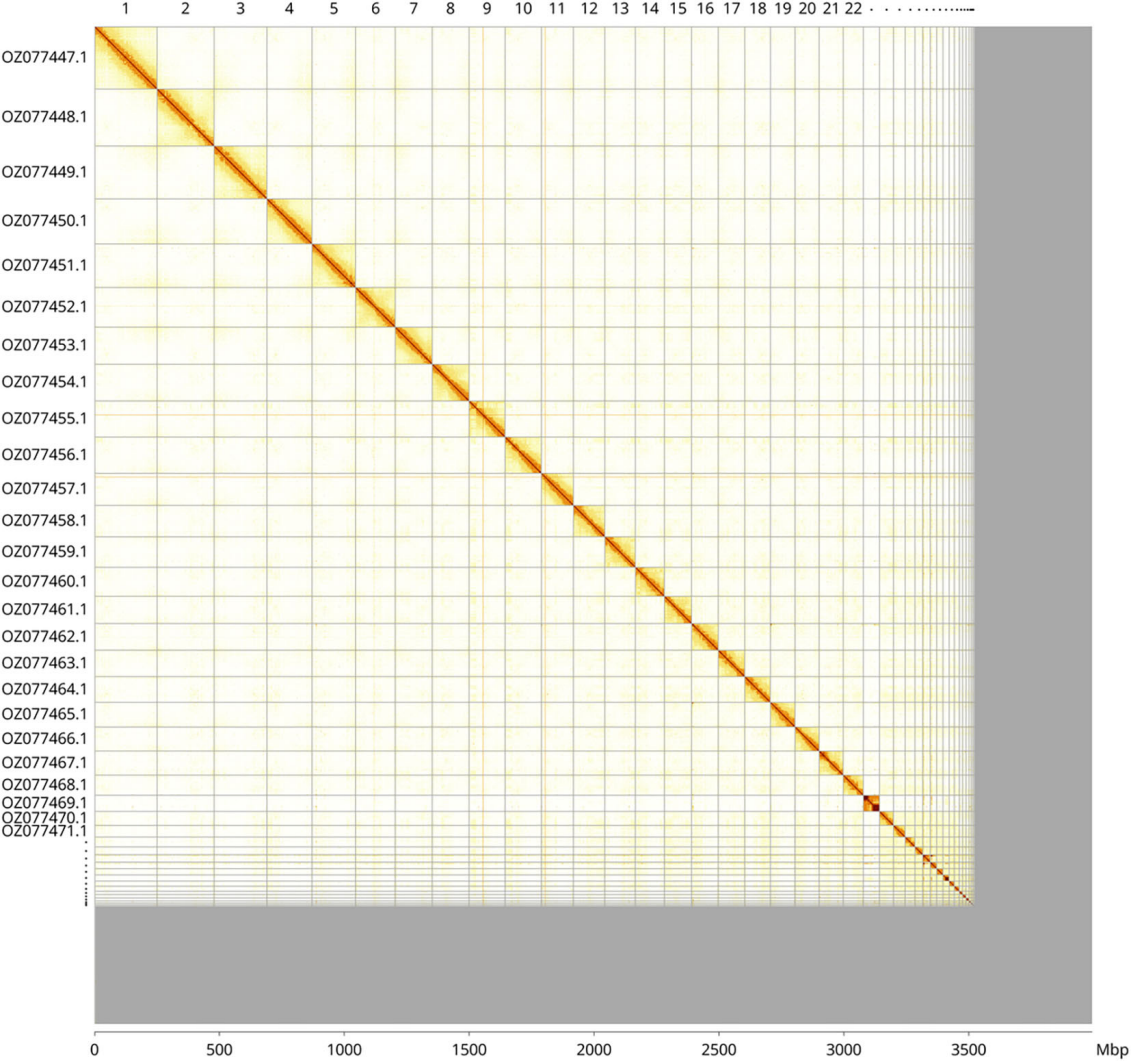
Hi-C contact map of the *Cetorhinus maximus* genome assembly. Assembled chromosomes are shown in order of size and labelled along the axes, with a megabase scale shown below. The plot was generated using PretextSnapshot.

Most of the haplotype 1 assembly sequence (88.16%) was assigned to 39 chromosomal-level scaffolds, representing 38 autosomes and the X sex chromosome. These chromosome-level scaffolds, confirmed by Hi-C data, are named according to size ( Figure 3; Table 3). Chromosome X was assigned by synteny to the genome assembly of *Carcharodon carcharias.*

**Table 3.** Chromosomal pseudomolecules in the haplotype 1 genome assembly of *Cetorhinus maximus* sCetMax3.

INSDC accession	Molecule	Length (Mb)	GC%
OZ077447.1	1	250.19	44.50
OZ077448.1	2	228.63	44
OZ077449.1	3	211.65	44.50
OZ077450.1	4	179.98	44.50
OZ077451.1	5	175.29	44.50
OZ077452.1	6	158.55	44
OZ077453.1	7	148.02	44
OZ077454.1	8	147.06	44
OZ077455.1	9	144.74	44.50
OZ077456.1	10	144.56	44
OZ077457.1	11	129.01	44.50
OZ077458.1	12	126.03	44
OZ077459.1	13	122.11	44.50
OZ077460.1	14	116.08	44
OZ077461.1	15	109.04	44
OZ077462.1	16	107.47	44
OZ077463.1	17	105.77	44
OZ077464.1	18	102.35	44
OZ077465.1	19	98.42	44
OZ077466.1	20	97.05	44.50
OZ077467.1	21	96.28	45
OZ077468.1	22	80.59	44
OZ077469.1	23	65.16	44.50
OZ077470.1	24	55.24	43.50
OZ077471.1	25	47.17	44
OZ077472.1	26	39.46	44
OZ077473.1	27	31.49	43.50
OZ077474.1	28	29.17	46.50
OZ077475.1	29	26.19	46.50
OZ077476.1	30	25.35	45
OZ077477.1	31	24.02	45
OZ077478.1	32	21.46	46.50
OZ077480.1	33	13.67	45
OZ077481.1	34	13.01	46.50
OZ077482.1	35	12.24	44.50
OZ077483.1	36	7.99	47.50
OZ077484.1	37	5.72	44
OZ077485.1	38	5.39	46.50
OZ077479.1	X	19.44	45

The mitochondrial genome was also assembled (length 16.67 kb, OZ077486.1). This sequence is included as a contig in the multifasta file of the genome submission and as a standalone record.

### Assembly quality metrics

**Figure 4.**
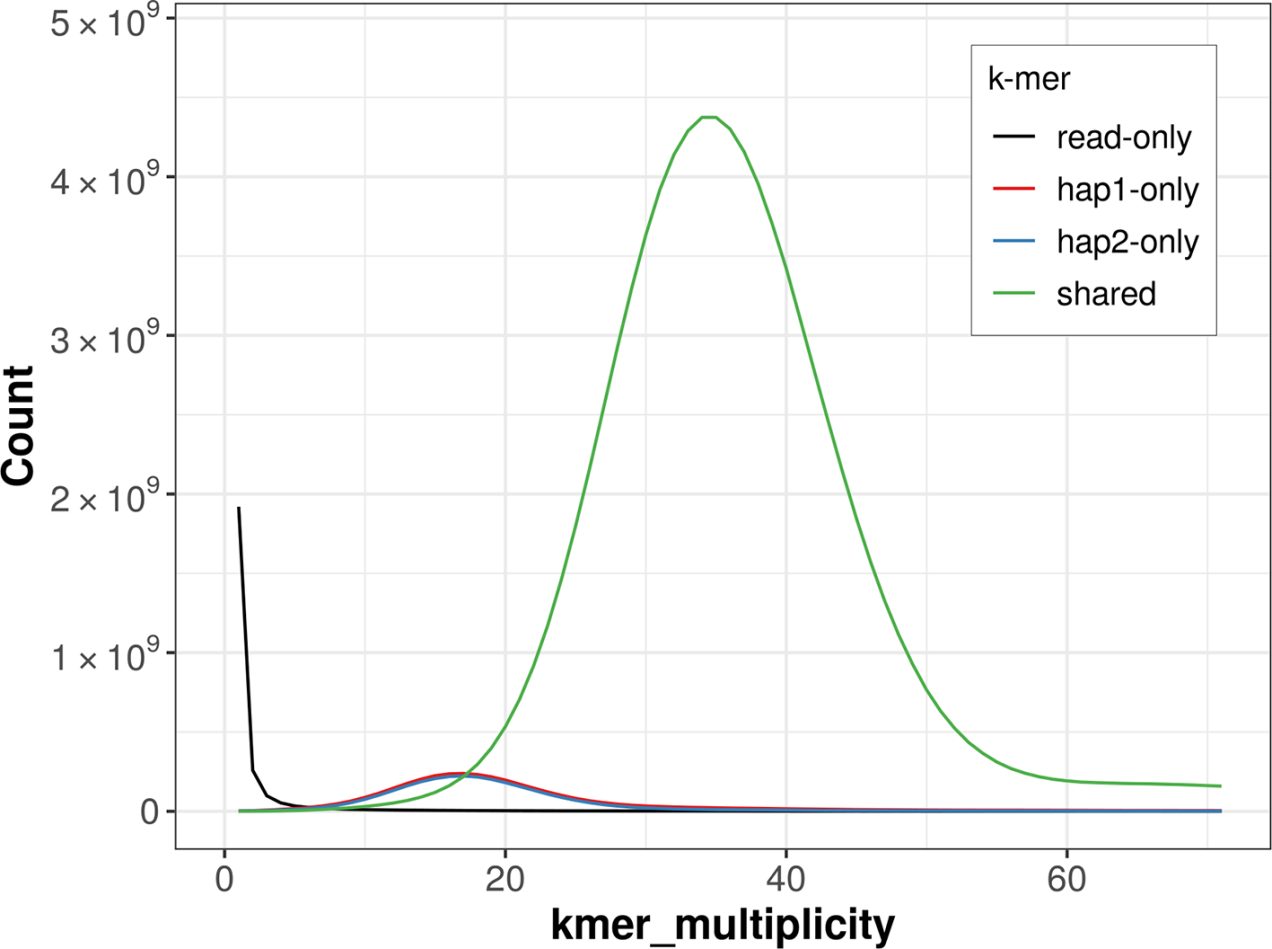
Evaluation of *k*-mer completeness using MerquryFK. This plot illustrates the recovery of *k*-mers from the original read data in the final assemblies. The horizontal axis represents *k*-mer multiplicity, and the vertical axis shows the number of *k*-mers. The black curve represents *k*-mers that appear in the reads but are not assembled. The green curve corresponds to *k*-mers shared by both haplotypes, and the red and blue curves show *k*-mers found only in one of the haplotypes.

For haplotype 1, the estimated QV is 60.1, and for haplotype 2, 60.4. When the two haplotypes are combined, the assembly achieves an estimated QV of 60.3. The *k*-mer completeness is 93.74% for haplotype 1, 92.83% for haplotype 2, and 99.56% for the combined haplotypes ( Figure 4).

**Figure 5.**
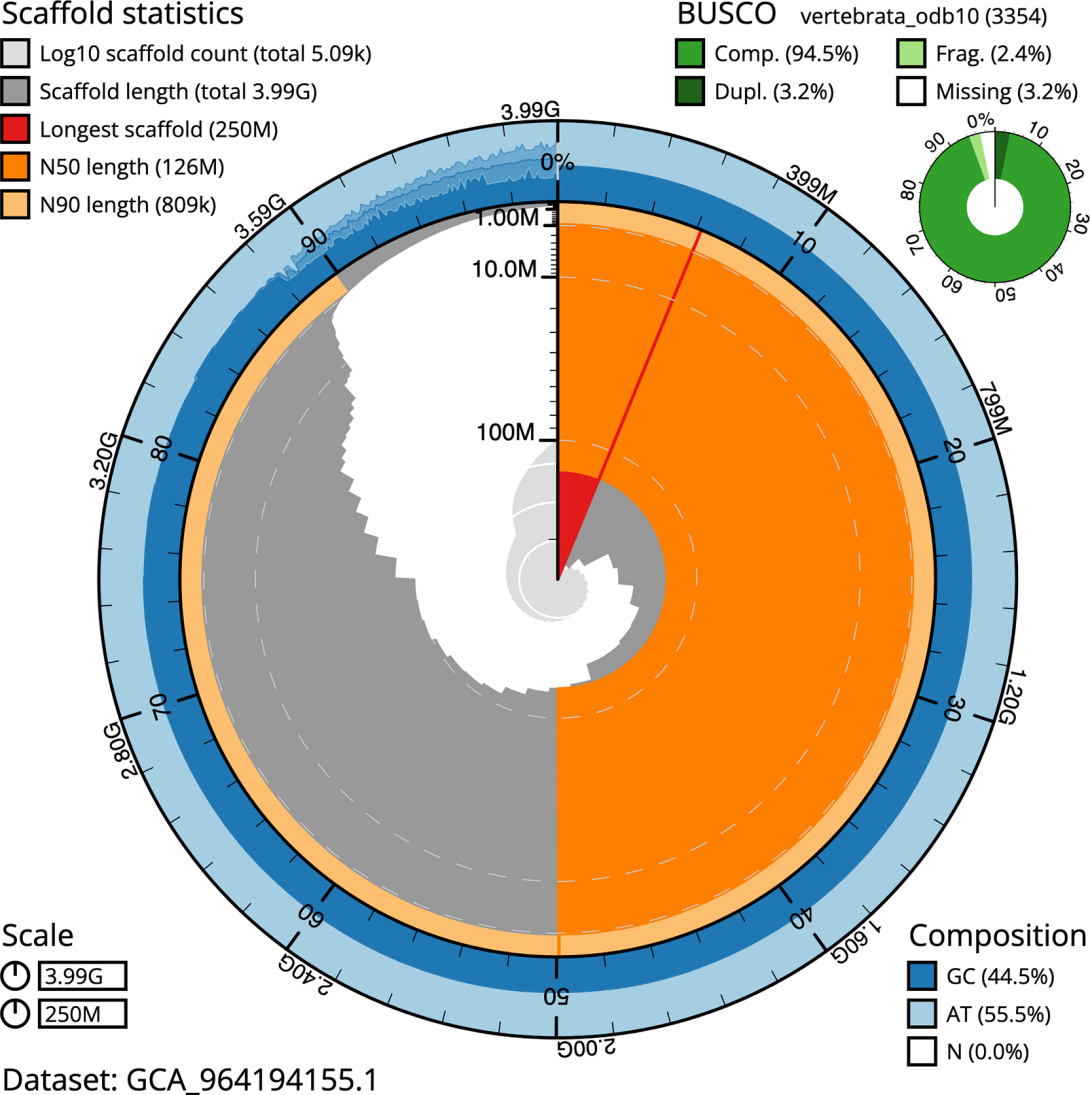
Assembly metrics for sCetMax3.hap1.1. The BlobToolKit snail plot provides an overview of assembly metrics and BUSCO gene completeness. The circumference represents the length of the whole genome sequence, and the main plot is divided into 1 000 bins around the circumference. The outermost blue tracks display the distribution of GC, AT, and N percentages across the bins. Scaffolds are arranged clockwise from longest to shortest and are depicted in dark grey. The longest scaffold is indicated by the red arc, and the deeper orange and pale orange arcs represent the N50 and N90 lengths. A light grey spiral at the centre shows the cumulative scaffold count on a logarithmic scale. A summary of complete, fragmented, duplicated, and missing BUSCO genes in the set is presented at the top right. An interactive version of this figure can be accessed on the BlobToolKit viewer.

**Figure 6.**
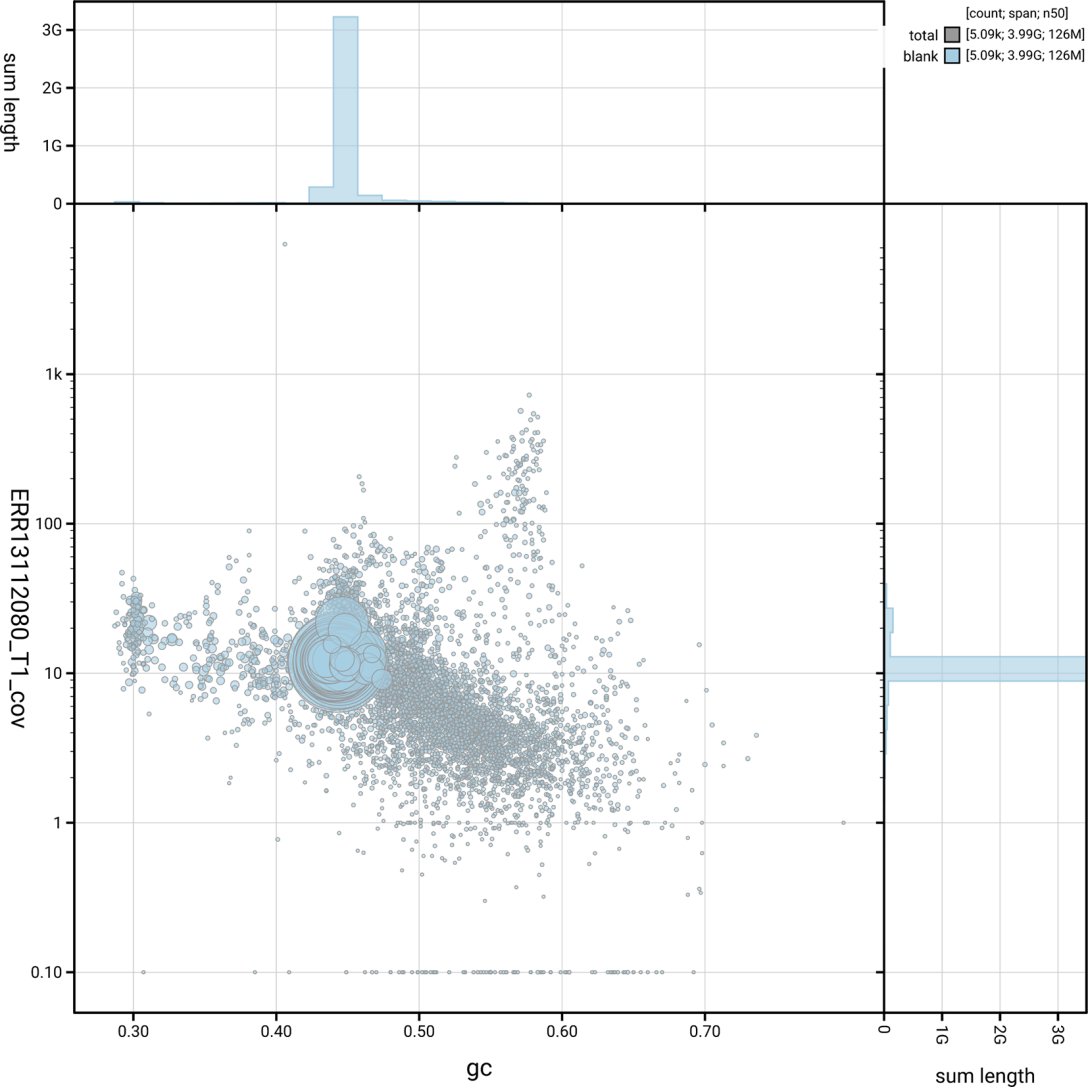
BlobToolKit blob plot for sCetMax3.hap1.1. The plot shows base coverage (vertical axis) and GC content (horizontal axis). The circles represent scaffolds, with the size proportional to scaffold length and the colour representing phylum membership. The histograms along the axes display the total length of sequences distributed across different levels of coverage and GC content. An interactive version of this figure is available on the BlobToolKit viewer.

BUSCO analysis using the metazoa_odb10 reference set ( *n* = 954) identified 97.4% of the expected gene set (single = 91.9%, duplicated = 5.5%) in haplotype 1. The snail plot in Figure 5 summarises the scaffold length distribution and other assembly statistics for haplotype 1. The blob plot in Figure 6 shows the distribution of scaffolds by GC proportion and coverage for haplotype 1.

Table 4 lists the assembly metric benchmarks adapted from Rhie *et al.* (2021) and the Earth BioGenome Project Report on Assembly Standards September 2024. The EBP metric, calculated for the haplotype 1, is **6.8.Q60**.

**Table 4.** Earth Biogenome Project summary metrics for the *Cetorhinus maximus* assembly.

Measure	Value	Benchmark
EBP summary (haplotype 1)	6.8.Q60	6.C.Q40
Contig N50 length	1.96 Mb	≥ 1 Mb
Scaffold N50 length	126.03 Mb	= chromosome N50
Consensus quality (QV)	Haplotype 1: 60.1; haplotype 2: 60.4; combined: 60.3	≥ 40
*k*-mer completeness	Haplotype 1: 93.74%; haplotype 2: 92.83%; combined: 99.56%	≥ 95%
BUSCO	C:97.4% [S:91.9%; D:5.5%]; F:2.0%; M:0.6%; n:954	S > 90%; D < 5%
Percentage of assembly assigned to chromosomes	88.16%	≥ 90%

**Notes:** EBP summary uses log10(Contig N50); chromosome-level (C) or log10(Scaffold N50); Q (Merqury QV). BUSCO: C = complete; S = single-copy; D = duplicated; F = fragmented; M = missing; n = orthologues.

## Author information

Contributors are listed at the following links:

• Members of the Wellcome Sanger Institute Tree of Life Management, Samples and Laboratory team
• Members of Wellcome Sanger Institute Scientific Operations – Sequencing Operations
• Members of the Wellcome Sanger Institute Tree of Life Core Informatics team
• Members of the Tree of Life Core Informatics collective
• Members of the Darwin Tree of Life Consortium

## Wellcome Sanger Institute – Legal and governance

The materials that have contributed to this genome note have been supplied by a Darwin Tree of Life Partner. The submission of materials by a Darwin Tree of Life Partner is subject to the **‘Darwin Tree of Life Project Sampling Code of Practice’**, which can be found in full on the Darwin Tree of Life website. By agreeing with and signing up to the Sampling Code of Practice, the Darwin Tree of Life Partner agrees they will meet the legal and ethical requirements and standards set out within this document in respect of all samples acquired for, and supplied to, the Darwin Tree of Life Project. Further, the Wellcome Sanger Institute employs a process whereby due diligence is carried out proportionate to the nature of the materials themselves, and the circumstances under which they have been/are to be collected and provided for use. The purpose of this is to address and mitigate any potential legal and/or ethical implications of receipt and use of the materials as part of the research project, and to ensure that in doing so we align with best practice wherever possible. The overarching areas of consideration are:

• Ethical review of provenance and sourcing of the material
• Legality of collection, transfer and use (national and international)

Each transfer of samples is further undertaken according to a Research Collaboration Agreement or Material Transfer Agreement entered into by the Darwin Tree of Life Partner, Genome Research Limited (operating as the Wellcome Sanger Institute), and in some circumstances, other Darwin Tree of Life collaborators.

## Data Availability

European Nucleotide Archive: Cetorhinus maximus (basking shark). Accession number PRJEB75718. The genome sequence is released openly for reuse. The *Cetorhinus maximus* genome sequencing initiative is part of the Darwin Tree of Life Project (PRJEB40665), the Sanger Institute Tree of Life Programme (PRJEB43745) and the Vertebrate Genomes Project (PRJNA489243). All raw sequence data and the assembly have been deposited in INSDC databases. The genome will be annotated using available RNA-Seq data and presented through the Ensembl pipeline at the European Bioinformatics Institute. Raw data and assembly accession identifiers are reported in Tables 1 and 2. Production code used in genome assembly at the WSI Tree of Life is available at https://github.com/sanger-tol . Table 5 lists software versions used in this study.
