# Supplementary material for: The genome sequence of the Basking Shark, Cetorhinus maximus (Gunnerus, 1765) (Lamniformes: Cetorhinidae)
Source: Wellcome Open Res. 2026 Mar 18;11:166. [Version 1] doi: 10.12688/wellcomeopenres.26114.1 (PMC13044535; doi:10.12688/wellcomeopenres.26114.1)
Supplement: Peer review history

# Reviewer report: Reviewer response for version 1

**DOI:** 10.21956/wellcomeopenres.28757.r151476

**Published:** 2026-04-02

**Authors:** L Filipe C Castro

**Affiliations:** 1 University of Porto, Porto, Portugal

A high-quality genome assembly for Cetorhinus maximus is reported. This is a valuable first genomic resource for a species in the family Cetorhinidae. The assembly stats support the conclusion that this is a robust and dependable reference genome.

 The manuscript is centered on genome production and assembly performance rather than on in-depth biological interpretation (as expected for a genome note). Yet, it will prove very useful for future work on elasmobranch evolution, comparative genomics, and conservation.

Are sufficient details of methods and materials provided to allow replication by others?

Yes

Is the rationale for creating the dataset(s) clearly described?

Yes

Are the datasets clearly presented in a useable and accessible format?

Yes

Are the protocols appropriate and is the work technically sound?

Yes

Reviewer Expertise:

Comparative genomics and Evolution

I confirm that I have read this submission and believe that I have an appropriate level of expertise to confirm that it is of an acceptable scientific standard.

# Reviewer report: Reviewer response for version 1

**DOI:** 10.21956/wellcomeopenres.28757.r151474

**Published:** 2026-04-01

**Authors:** Bingpeng Xing

**Affiliations:** 1 Third Institute of Oceanography Ministry of Natural Resources, Xiamen, Fujian, China

This manuscript presents the first publicly available genome assembly for Cetorhinus maximus and, to my knowledge, the first genomic resource for Cetorhinidae. Overall, the data support the main conclusion that this study provides a valuable reference genome resource.

 However, several points would benefit from revision.

 First, the Introduction is informative but could better explain the genomic significance of this resource. At present, it focuses more on species biology and conservation status than on why this genome is especially important for future studies of elasmobranch evolution, comparative genomics, or conservation genomics.

 Second, the methods are generally appropriate and consistent with current practice for high-quality vertebrate genome assembly. The workflow using PacBio HiFi, Hi-C scaffolding, manual curation, and standard quality assessment tools is sound. Still, a few details could be clarified, especially how haplotype-resolved outputs were handled before scaffolding and what main evidence supported the manual breaks and joins during curation.

 Third, the assembly metrics are strong overall, but the manuscript should acknowledge more explicitly that a few values are slightly below ideal benchmark targets. For example, 88.16% of the primary assembly was assigned to chromosomes, which is very good but slightly below the commonly cited >90% threshold. Similarly, BUSCO duplication and haplotype-specific k-mer completeness could be interpreted more transparently. These are not major weaknesses, but they should be discussed briefly.

 Finally, the Results/Discussion are clear but somewhat descriptive. Even for a genome note, the manuscript would benefit from a few sentences placing this assembly in a broader comparative context, such as how its size or structure compares with other lamniform or elasmobranch genomes.

Are sufficient details of methods and materials provided to allow replication by others?

Yes

Is the rationale for creating the dataset(s) clearly described?

Yes

Are the datasets clearly presented in a useable and accessible format?

Yes

Are the protocols appropriate and is the work technically sound?

Yes

Reviewer Expertise:

genome，marine biodiversity，DNA barcoding，mitogenome

I confirm that I have read this submission and believe that I have an appropriate level of expertise to confirm that it is of an acceptable scientific standard.

# Reviewer report: Reviewer response for version 1

**DOI:** 10.21956/wellcomeopenres.28757.r151467

**Published:** 2026-03-25

**Authors:** Alejandro Mechaly

**Affiliations:** 1 Instituto de Investigaciones en Biodiversidad y Biotecnología (INBIOTEC-CONICET), Mar del Plata, Argentina

This manuscript presents a high-quality genome assembly of *Cetorhinus maximus*, which, to the best of my knowledge, is the first genome available for a member of the family Cetorhinidae. The sequencing strategy and assembly approach are appropriate and consistent with current standards, and the assembly metrics (e.g., scaffold N50, BUSCO completeness, and QV ~60) indicate that this is a robust and reliable reference genome.

 Considering the scope of a genome note, the manuscript is appropriately focused on data generation and assembly quality rather than extensive biological analyses. In this sense, it achieves its main goal and provides a useful resource for future studies on elasmobranch biology, evolution, and conservation.

Are sufficient details of methods and materials provided to allow replication by others?

Yes

Is the rationale for creating the dataset(s) clearly described?

Yes

Are the datasets clearly presented in a useable and accessible format?

Yes

Are the protocols appropriate and is the work technically sound?

Yes

Reviewer Expertise:

Fish reproduction and endocrinology

I confirm that I have read this submission and believe that I have an appropriate level of expertise to confirm that it is of an acceptable scientific standard.
